# Reducing Broad-Spectrum Antibiotic Treatment of Simple Group A Streptococcal Infections to Reduce Harm to the Microbiome

**DOI:** 10.7759/cureus.15629

**Published:** 2021-06-13

**Authors:** Tyrone J Sumibcay, Jannet J Lee-Jayaram, Loren G Yamamoto

**Affiliations:** 1 Pediatrics, Kapi`olani Medical Center for Women & Children, Honolulu, USA; 2 Pediatric Emergency Medicine, University of Hawai'i, John A. Burns School of Medicine, Honolulu, USA

**Keywords:** penicillin, microbiota, streptococcal pharyngitis, microbiome, broad-spectrum antibiotics

## Abstract

Background

Broad-spectrum antibiotics disrupt the human microbiome resulting in a greater risk of harmful, long-term conditions that impact human health. Group A streptococcal (GAS) infections can be treated with penicillin.

Objective

We examined the treatment of simple GAS infections to assess the use of broad-spectrum antibiotics.

Methods

Smart relational database extraction queries from January 1, 2016 to July 10, 2019 (3.6 years) of patients less than 22 years old in a 4-hospital system electronic medical record (EMR).

Results

We found 1778 non-ED outpatients and 873 ED patients with simple GAS infections who were not allergic to penicillin. A total of 75% and 44% of non-ED and ED patients were treated with broad-spectrum antibiotics, respectively (p < 0.001). Older patients were treated with penicillin alone more frequently than younger age groups (p < 0.001).

Conclusion

These findings highlight opportunities for clinicians to reduce the utilization of broad-spectrum antibiotics for the treatment of simple GAS infections to reduce harm to the microbiome.

## Introduction

The human microbiome is the microbial community that is crucial for human health and functioning. Disruption of the human microbiome, caused by a reduction in the diversity of its microorganisms that occurs with antibiotic treatment, can play a role in the development of harmful, long-term conditions that impact human health. Narrow-spectrum antibiotics disrupt the microbiome less than broad-spectrum antibiotics [1-5]. Antibiotic treatment of Group A streptococcal (GAS) infections, serves as an area where harm to the microbiome can be reduced since it can be treated with the narrow-spectrum antibiotic, penicillin, with 100% sensitivity. However, clinicians frequently prescribe broader spectrum antibiotics such as amoxicillin, cephalosporins, and clindamycin. While some might not consider amoxicillin to be a broad-spectrum antibiotic, its coverage is clearly broader than that of penicillin and it is commonly cited as a common cause of pseudomembranous colitis along with clindamycin [6,7,8,9]. The purpose of this study is to characterize the antibiotics used to treat simple streptococcal throat infections and scarlet fever in children and to assess the degree of unnecessary broad-spectrum antibiotic use for these conditions.

Preliminary abstract of this work was presented at the 2019 Western Medical Research Conference in Carmel, CA on January 23, 2020. This abstract was published in the Journal of Investigative Medicine. An abstract of this work was submitted to the May 2020 Pediatric Academic Societies Meeting in Philadelphia, where it was accepted for presentation; however, the meeting was canceled due to COVID-19.

## Materials and methods

We performed a retrospective data extraction from the electronic medical record (EMR) system of a 4-hospital health care system from January 1, 2016 to July 10, 2019 (3.6 years) of patients who were less than 22 years old at the time of the encounter. This was accomplished by performing a stepwise smart relational database query from the EMR to extract all outpatient (office, clinic, acute care, urgent care, ED) encounters with International Classification of Diseases, 10th Revision, Clinical Modification (ICD-10-CM) diagnosis codes of acute streptococcal pharyngitis/tonsillitis or scarlet fever. This query included demographic information, disposition, medication allergies, medications (which included medications administered during the encounter, medications prescribed at the encounter, and medications prescribed within four days after the encounter), all encounter diagnoses, and all problem list diagnoses. Antibiotics were classified into groups according to the treatments administered during the encounter and prescribed within the next four days, which permitted us to capture changes in treatment in the four days immediately after the encounter. In some instances, a rapid streptococcal test could have been negative, but if this was followed by a positive culture result two days later, a prescription ordered within the four-day period was captured by this data query. The antibiotic classification was based on the broadest spectrum antibiotic administered or prescribed for the encounter with the assumption that the broadest spectrum drug harms the microbiome the most. The broadness sequence used was the following: amoxicillin < azithromycin < trimethoprim/sulfamethoxazole (TMP/SMX) < cephalosporins < amoxicillin/clavulanate < clindamycin. While this sequence is debatable, the number of prescriptions for azithromycin and TMP/SMX was low. If the patient was given intramuscular benzathine penicillin and prescribed amoxicillin, this encounter was classified as treatment with amoxicillin. If the patient was given intramuscular ceftriaxone but treated orally with penicillin, this encounter was classified as treatment with a cephalosporin. 

Our target population was patients with simple streptococcal pharyngitis, tonsillitis, or scarlet fever (mild GAS infections). Thus, we excluded patients who were hospitalized during this encounter or left against medical advice. Patients were also excluded if they had a penicillin allergy or had co-infections or comorbidities that would require the use of broad-spectrum antibiotics, such as otitis media, sinusitis, impetigo, abscess, lymphadenitis, chronic/recurrent streptococcal infections, pneumonia, cellulitis, burns, urinary tract infection, renal disease (other than minimal change disease and microscopic hematuria), concurrent long-term corticosteroid treatment, indwelling catheters, cancers, congenital heart disease, valvular heart disease, chronic diseases associated with immunosuppression or bacterial infection recurrence.

## Results

There were 3,632 encounters that met our initial search criteria in which the encounter diagnosis contained an ICD-10-CM code of streptococcal pharyngitis/tonsillitis or scarlet fever. We separated the groups into ED and non-ED (office, primary care, clinic, acute care, urgent care). The exclusions and age/gender distributions of the remaining encounters are described in Table 1. Penicillin allergy was more common in the non-ED group compared to the ED group (9.1% versus 5.5%, p = 0.0002). Other exclusions were less common in the non-ED group compared to the ED group (18% versus 22%, p = 0.001).

**Table 1:** Cohort demographics (3632 encounters).

	All	0 to 3 years	4 to 12 years	13 to 21 years
Non-ED	2424	263 (11%)	1467 (61%)	694 (29%)
Penicillin allergic	220 (9.1%)	15	147	58
Other exclusions	426 (18%)	91	253	82
Male inclusions	918	80	559	279
Female inclusions	860	77	508	275
ED	1208	103 (9%)	761 (63%)	344 (28%)
Penicillin allergic	67 (5.5%)	6	43	18
Other exclusions	268 (22%)	29	162	77
Male inclusions	438	37	305	96
Female inclusions	435	31	251	153

Table 2 summarizes the antibiotic treatment groups for the cohort into penicillin (narrow spectrum), broad-spectrum antibiotics, and no antibiotic treatment (despite a diagnosis of GAS pharyngitis/tonsillitis or scarlet fever in the encounter diagnosis). Non-ED patients were treated with broad-spectrum antibiotics more often than the ED patients (75% versus 44%, p < 0.0001). In comparing the age groups of the combined cohort, the 0 to 3, 4 to 12, and 13 to 21-year-old age groups were treated with penicillin alone in 22%, 27%, and 37% of the encounters, respectively (p < 0.0001).

**Table 2:** Antibiotic treatment groups by age group. Long-acting benzathine penicillin (benzPCN) was mostly given as monotherapy in the penicillin group, but sometimes it was given concurrently with penicillin and broad-spectrum oral antibiotics. These numbers are in subset brackets { } because they are a subset of and included in the column to its left.

	Penicillin	{benzPCN}	Broad spectrum	{Broad spectrum + benzPCN}	None
Non-ED	362 (20%)	{227}	1337 (75%)	{30}	79 (4%)
0 to 3 years	25	{22}	124	{5}	8
4 to 12 years	179	{124}	843	{14}	45
13 to 21 years	158	{81}	370	{11}	26
ED	419 (48%)	{329}	380 (44%)	{50}	74 (8%)
0 to 3 years	24	{19}	34	{2}	10
4 to 12 years	254	{205}	253	{28}	49
13 to 21 years	141	{105}	93	{20}	15
Combined groups	781 (29%)	{556}	1717 (65%)	{80}	153 (6%)
0 to 3 years	49	{41}	158	{7}	18
4 to 12 years	433	{329}	1096	{42}	94
13 to 21 years	299	{186}	463	{31}	41

Table 3 summarizes the broad-spectrum antibiotic groups prescribed. Within this group, amoxicillin comprised 88% of the non-ED patients, and 79% of the ED patients (p = 0.005). The other antibiotics were used less frequently, but it was noticeable that azithromycin was prescribed 7.3% in the non-ED patients, and 3.7% in the ED patients (p = 0.02). 

**Table 3:** Broad-spectrum antibiotic treatment groups by age group. TMP/SMX: Trimethoprim/sulfamethoxazole.

	Amoxicillin	Cephalosporins	Amoxicillin/Clavulanate	Azithromycin	Clindamycin	TMP/SMX
Non-ED	1172	37	28	97	0	3
0 to 3 years	114	3	1	6	0	0
4 to 12 years	754	20	12	55	0	2
13 to 21 years	304	14	15	36	0	1
ED	301	37	9	14	19	0
0 to 3 years	26	6	0	1	1	0
4 to 12 years	214	18	5	8	8	0
13 to 21 years	61	13	4	5	10	0
Combined groups	1473	74	37	111	19	3
0 to 3 years	140	9	1	7	1	0
4 to 12 years	968	38	17	63	8	2
13 to 21 years	365	27	19	41	10	1

## Discussion

Our data indicate that amoxicillin and other broad-spectrum antibiotics are commonly used to treat simple GAS infections. Since penicillin has a narrow spectrum and GAS is 100% sensitive to penicillin, broad-spectrum antibiotic treatment is not necessary to treat GAS and it results in greater harm to the microbiome. 

Interestingly, 6% of the cohort was not treated with antibiotics. It is known that simple GAS pharyngitis infections will usually resolve without antibiotic treatment. Prompt antibiotic treatment, however, shortens the clinical course of symptoms and reduces the risk of suppurative complications, contagiousness, and acute rheumatic fever [10].

Study limitations include the retrospective nature of the study which makes the identification of simple GAS infections difficult to confirm; however, the methodology of reviewing all ICD-10-CM encounter diagnoses and the problem list should have identified complicated cases most of the time (Table 1). In 13 instances (eight non-ED and five ED), a case in which a patient who was initially treated with penicillin for a simple GAS infection was given a new prescription for a broad-spectrum antibiotic, which might have been for a clinically valid reason (e.g., otitis media); however, we did not review the clinical encounter data for the subsequent prescriptions which could have been done after all the data was collated and summarized but it was not part of the initially approved protocol to enter the patient's chart. Such encounters could have been simply removed since they are not simple GAS infections; however, such patients sustained the broad-spectrum antibiotic’s harm to their microbiome, and thus, we classified such patients as broad spectrum. Although it could be that the study group contains some patients who were treated with broad-spectrum antibiotics based on clinical necessity, it is more than likely that most of these patients could have been treated with penicillin.

Penicillin allergies are very likely to be reliable from a medical history standpoint using this methodology; however, it is well known that most patients who report a history of penicillin allergy are not truly allergic. Such patients subject themselves to less effective antibiotics (e.g., macrolides) and antibiotics resulting in greater harm to the microbiome (e.g., clindamycin, cephalosporins) [11].

Penicillin can be given orally four times per day (QID) or as a single intramuscular injection of long-acting benzathine penicillin. The 1.2 million units of long-acting benzathine penicillin can currently be purchased for $313, while 40 500-mg penicillin VK tablets (a 10-day course) can be purchased for $6, and the 200-ml bottle of 250 mg/5 ml penicillin VK can be purchased for $12. Thirty 500 mg amoxicillin tablets can be purchased for $6 and the 100-ml bottle of 400 mg/5 ml amoxicillin can be purchased for $5 [12].

Long-acting benzathine penicillin injections were given frequently in both the non-ED and the ED cohorts. Of the 362 non-ED and the 419 ED patients treated with penicillin alone, 227 (63%) and 329 (79%) were given benzathine penicillin, respectively. Of the 1337 non-ED patients and 380 ED patients treated with broad-spectrum antibiotics, 30 and 50 were also given benzathine penicillin, respectively. Benzathine penicillin must be refrigerated, and although it could be pre-purchased by office practices, it is generally obtained from a pharmacy.

Benzathine crystalline penicillin intramuscular injections are painful [10]. They are also viscous and opaque increasing the risk of intravascular injection [13]. Benzathine penicillin is known to be effective, but its duration of roughly 14 days or longer [14] exceeds the standard 10-day treatment duration. Prolonged or excessively long durations of antibiotics, even if it is a narrow-spectrum one, can prolong the adverse effect on the microbiome. In patients who are anticipated to complete a 10-day course of oral penicillin sufficiently, the additional pain, risk, and high cost of long-acting benzathine penicillin should be reconsidered.

Amoxicillin has the advantage of three times per day (TID) dosing, and some clinicians will dose this twice per day (BID). Cephalexin is usually dosed QID, in which case there would be no dosing advantage over oral penicillin. Azithromycin is dosed once daily. Amoxicillin/Clavulanate and clindamycin are dosed TID or BID, but their main indication for treating GAS throat infections is for suspected recurrent GAS infections or a GAS carrier state. If these conditions were described in the problem list or the encounter diagnosis, then these cases should have been excluded. 

While GAS is 100% sensitive to penicillins and cephalosporins, there is growing resistance to macrolides (azithromycin) and clindamycin. Additionally, GAS sometimes possesses inducible macrolide-lincosamide-streptogramin (MLS) resistance [15]. 

QID dosing for penicillin is a clinical disadvantage that must be weighed against its advantage of reducing harm to the microbiome. Liquid penicillin has the reputation of poor taste compared to the other antibiotics that are used, but this problem with taste does not apply to pills, tablets, and capsules. This factor might be the reason for the higher penicillin frequency in the older patients who can swallow pills, compared to the younger patients who require liquid antibiotic formulations. The clinical practice of prescribing broad-spectrum antibiotics for GAS infections evolved in an era during which the importance of the microbiome was not appreciated as much as it is today. Now with its importance identified, there should be a better understanding and appreciation of the harm resulting from unnecessary broad-spectrum antibiotics. While some consider amoxicillin to be a standard treatment for simple GAS infection, this should be reconsidered since its coverage is unnecessarily broad.

## Conclusions

Our findings highlight opportunities for clinicians to reduce the utilization of broad-spectrum antibiotics for the treatment of simple GAS infection to reduce harm to the microbiome, which is essential in maintaining human health. GAS is still 100% sensitive to penicillin which is available orally, and for patients anticipated to have issues with compliance/adherence, single-dose long-acting penicillin injection is a treatment option. Since most broad-spectrum antibiotic-treated patients were treated with amoxicillin and cephalosporins, changing to narrow-spectrum penicillin is a relatively simple practice change that most patients can tolerate while reducing harm to the microbiome.
